# Advances in vaccine development for *Chlamydia trachomatis*

**DOI:** 10.1093/femspd/ftae017

**Published:** 2024-07-23

**Authors:** Taylor B Poston

**Affiliations:** Department of Pediatrics, University of North Carolina at Chapel Hill, Chapel Hill, NC 27599, United States

**Keywords:** vaccine, T cell, adjuvant, *Chlamydia*, antibody

## Abstract

*Chlamydia trachomatis* is the most prevalent bacterial sexually transmitted infection globally. Antibiotic treatment is highly effective, but infection is often asymptomatic resulting in most individuals going undetected and untreated. This untreated infection can ascend to the upper female genital tract to cause pelvic inflammatory disease, tubal factor infertility, and ectopic pregnancy. *Chlamydia* screening and treatment programs have failed to control this epidemic and demonstrate the need for an efficacious vaccine to prevent transmission and disease. Animal models and human epidemiological data reveal that natural immunity can provide partial or short-lived sterilizing immunity. These data further demonstrate the importance of eliciting interferon gamma (IFNγ)-producing cluster of differentiation 4 (CD4) T cells (Th1 and Th1/17 cells) that can likely synergize with antibody-mediated opsonophagocytosis to provide optimal protection. These studies have guided preclinical rational vaccine design for decades and the first Phase 1 clinical trials have recently been completed. Recent advances have led to improvements in vaccine platforms and clinically safe adjuvants that help provide a path forward. This review describes vaccine models, correlates of immunity, antigen and adjuvant selection, and future clinical testing for *Chlamydia* vaccine development.

## Teaser

This review summarizes recent developments in mucosal and *Chlamydia* immunity applicable to *Chlamydia* vaccine design.

## Introduction

*Chlamydia trachomatis* (CT) is a gram-negative obligate intracellular bacterium that infects the ocular and genital tract epithelium. Serovars A, B, Ba, and C cause ocular trachoma, and sexually transmitted genital infection and associated diseases are caused by serovars D–K, with serovars L1–L3 causing lymphogranuloma venereum (LGV). The developmental cycle is made up of the elementary body (EB) form that attaches and infects the epithelial target cell and then differentiates into the reticulate body (RB) form that replicates in an intracellular inclusion. The RBs differentiate back to infectious EBs, which are released from the host cell to begin another round of infection. Genital CT infection and associated diseases are a significant global health burden with ∼131 million new cases occurring each year (Newman et al. 2015). In the United States, >1.8 million people are infected annually (Geisler et al. 2022) and direct treatment costs exceed $500 M (Owusu-Edusei et al. 2013). Antibiotic treatment is highly effective but because > 70% of infections are asymptomatic (Stamm 1999, Farley et al. 2003), many infected persons go undetected and untreated. In women, CT infects the endocervix but can spread to the endometrium and oviducts to cause symptomatic (∼10%) or subclinical (∼25%) pelvic inflammatory disease (PID). Sequelae include chronic pelvic pain (30%), infertility (10%), and ectopic pregnancy (10%) (Haggerty et al. 2010). CT enhances the acquisition of other STIs and is an independent risk factor for cervical cancer (Chan et al. 2016). Prior CT infection increases the risk for fallopian tube carcinomas and epithelial ovarian cancer. In men, urethritis, epididymitis, and orchitis are the most common acute manifestations (Mackern-Oberti et al. 2017), and while long term-sequelae are not associated with CT genitourinary infection in males, proctitis, prostatitis, and prostate cancer have been linked to CT, particularly in men who have sex with men (MSM) (Bryan et al. 2020). Also, among MSM, 10%–15% test positive for rectal *Chlamydia* at sexual health clinics and the US Centers for Disease Control and Prevention recommends at least annual screening in MSM individuals engaging in receptive anal sex (Khosropour et al. 2021). LGV infections are also increasing and can result in highly debilitating chronic sequelae like bubo formation, fistulas, fibrosis, and rectal stenosis (Williamson and Chen 2020). Despite screening programs, infection rates continue to rise (Shannon and Klausner 2018). Barrier methods of contraception like condom use are effective but utilization rates are low (Bearinger et al. 2007). There is a clear and urgent need for a vaccine that combats this ongoing epidemic and associated complications, which will likely require immunization of both sexes to prevent transmission.

### Models of CT genital infection

Chlamydiae infect a wide range of species, but productive infection is generally host-specific, with significant limitations to using CT in mice. CT serovar D shedding is detected for only 10 days in immunologically normal mice after vaginal inoculation (O'Connell et al. 2011), and the infection course is unaltered in mice lacking CD4 T cells (Morrison et al. 2011) suggesting CT delivered intravaginally is insufficient for assessing adaptive immune responses. Transcervical inoculation of mice with human strains results in more productive infections (Rajeeve and Sivadasan 2020), but bypasses the cervix, a significant mucosal and immunological barrier to the upper genital tract. Transcervical inoculation with human serovars induces minimal oviduct pathology, presenting another limitation to this model. Thus, preclinical studies in mice frequently use the natural mouse pathogen *Chlamydia muridarum* (CM) since CT is highly susceptible to murine IFNγ-induced GTPases (Nelson et al. 2005). Female mice inoculated vaginally with CM recapitulate immune mediators of protection and fallopian tube pathology observed with severe CT infection in cisgender women. This pathology, specifically oviduct scarring and fibrosis with post-obstructive dilatation or hydrosalpinx, provides a robust model for vaccine testing. The female mouse model has been extensively utilized in the field, while the male and male-to-female sexual transmission model has gained focus over the last 10 years (Sobinoff et al. 2015, O'Meara et al. 2016, Pal et al. 2019, Bryan et al. 2020, Bryan et al. 2023). Additionally, CM stock populations contain sub-populations of genetic variants expressing phenotypic differences (Ramsey et al. 2009). Inoculation of mice with different plaque-purified clonal isolates from these stocks results in infections of varying severity (Poston et al. 2018). Ideally, a clone selected for vaccine studies should reflect the infection profile and pathology of its multiclonal parent stock, because it yields consistent levels of shedding and pathology between individual mice.

The female guinea pig model using the natural pathogen *Chlamydia caviae* strain provides a second rodent model. Genital tract disease closely resembles that of women, but the lack of immunological reagents is a significant barrier to vaccine studies (Rank and Whittum-Hudson 2010). The pig model utilizing *Chlamydia suis* is being explored as a large animal vaccine model due to the reproductive cycle and genital tract anatomy being like humans, but reagents are also less available compared to mice and specialized veterinary expertise is often needed due to logistical difficulties associated with infecting large animals (Lorenzen et al. 2015, Amaral et al. 2020). Non-human primates continue to serve as major preclinical vaccine testing models prior to phase 1 testing of vaccines in humans. This challenge model is limited for CT due to ocular and genital tract infections being shorter and self-limited, compared to humans, but provides utility for assessment of vaccine-elicited T and B cell responses to identify correlates of protection.

### Correlates of chlamydial immunity

Studies of natural immunity to CT in humans indicate the importance of T cells for CT infection resolution and protection from reinfection. Women with confirmed prior infection had lower chlamydial loads when reinfected (Batteiger et al. 2010). Women who spontaneously cleared infection without treatment exhibited increased CT-specific IFNγ+ CD4 T cells and were less likely to be reinfected when compared to women who required antibiotics for infection resolution (Geisler et al. 2013). HIV-infected women with low CD4 counts experienced higher rates of chlamydial PID (Kimani et al. 1996), and CT-specific IFNγ+ CD4 T cells are associated with resistance to reinfection (Russell et al. 2016). In contrast, among a cohort of highly CT-exposed women, serum and cervical anti-CT IgG correlated with increased risk of incident infection during a year of follow-up; with hazard ratios increasing 3.6-fold and 22.6-fold with each unit of serum and cervical IgG, respectively (Darville et al. 2019). IFNγ secreting CD4 T cells (Th1 and Th1/17 cells) are critical players in the protective immune response to this obligate intracellular bacterium that replicates within a protective vacuole inside epithelial cells (Darville 2021). CD4 T cell immunity is enhanced in the presence of chlamydial-specific IgG (Farris et al. 2010) by accelerating myeloid cell-mediated killing through opsonization (Naglak et al. 2016) and by enhanced Fc-mediated antigen presentation (Moore et al. 2003). IFNγ is needed for optimal antibody-mediated immunity against murine genital chlamydial infection (Naglak et al. 2016). Evidence for effective *in vivo* neutralization of chlamydial entry is minimal, potentially reflecting redundancy in adhesin-ligand interactions. Women with the highest anti-CT IgG responses may be Th2-skewed, resulting in inadequate IFNγ responses. Previous data reinforce the importance of eliciting robust and appropriate functional CD4 T cell responses, generating a milieu that enables antibody-mediated opsonophagocytosis (Naglak et al. 2017). Vaccination of male and female mice revealed a similar interplay where memory CD4 T cells in females and antibodies in male ejaculate synergized to elicit sterilizing immunity against transmission, suggesting vaccination of both sexes could be ideal for reducing infection prevalence in humans (O'Meara et al. 2016).

Short-lived sterilizing immunity after infection is observed in animal models (Rank and Whittum-Hudson 2010). Gene-knockout, depletion, and adoptive transfer studies in mice consistently demonstrate that CD4 T cells are the primary mediators of immunity (Perry et al. 1997, Wang et al. 1999, Stary et al. 2015) and that polyfunctional Th1 cells (IFNγ+ TNFα+ interleukin (IL)-2+) secreting high cytokine levels correlate with protection (Yu et al. 2011, Poston et al. 2017). These studies have focused on immune responses in the female reproductive tract, but male mouse studies have also demonstrated the importance of CD4 T cells in clearing infection (Cunningham et al. 2010, Sobinoff et al. 2015, O'Meara et al. 2016). Most activated CD4 T cells are not terminally differentiated and may shift between subsets to alter their cytokine profile (i.e. Th17–Th1 shift) or exhibit properties of multiple subsets where a more extensive range of cytokines are produced (i.e. bifunctional Th1/17) (Zielinski et al. 2012, Wacleche et al. 2016). Th1 or Th1/17 cells produce IFNγ that induces cellular responses that starve intracellular chlamydiae of tryptophan (Thomas et al. 1993, Nelson et al. 2005, Leonhardt et al. 2007) and enhance phagocyte effector functions (Al-Zeer et al. 2013, Tietzel et al. 2019). IL-17A independently promotes epithelial integrity and induces the production of antimicrobial peptides (Mills 2023). Vaccine-induced IL-17A is important for protection against other mucosal bacterial pathogens (Lin et al. 2010) and higher levels of IL-17 induced by a subunit outer membrane protein vaccine have been linked to lower chlamydial burden in mice and less disease upon challenge (Lizarraga et al. 2020). In CT-exposed women, Th17-lineage cells were significantly increased in those who did not experience follow-up CT infection compared to those who did, suggesting an important role for these cells in protection (Yount et al. 2023).

The role of CD8 T cells against *Chlamydia* infection is less clear. Mouse models reveal they are dispensable for clearance. Yet, adoptively transferred chlamydia-specific CD8 T cell clones can migrate to the infected genital tract and enhance clearance through the production of IFNγ (Igietseme et al. 1994). Human studies have correlated the frequency of antigen-specific CD8 T cells with limiting infection to the cervix (Russell et al. 2016). This could implicate a role in preventing ascension to the upper genital tract and the development of disease. However, the production of TNF by *Chlamydia*-specific CD8 T cells during infection of naïve mice has been shown to play a role in immunopathology (Murthy et al. 2011, Vlcek et al. 2016). Recent data reveal this may be a phenomenon restricted to the intravaginal challenge of naïve mice since vaccine-elicited antigen-specific CD4 T cells were able to prevent this pathological response (Murthy et al. 2024).

T cells can traffic from the circulation or respond directly in the infected tissue as a tissue-resident memory (TRM) population. Adjuvants and routes of immunization that induce Th1 or Th1/17 cells that traffic to the genital tract will promote vaccine efficacy. Intranasal vaccination of female mice with inactivated *Chlamydia* complexed to nanoparticles with the toll-like receptor (TLR)7/8 agonist resiquimod induced IFNγ+ TRM CD4 T cells in the genital tract (Stary et al. 2015). Mice vaccinated subcutaneously were unprotected despite generating a systemic T cell response, demonstrating an essential role for non-circulating TRM in mice. A prevailing hypothesis is that mucosal immunization or infection induces plastic Th17 cells that migrate to infected tissue where they differentiate into Th1 (ex-Th17) or Th1/17 cells that become persistent tissue-resident cells capable of responding rapidly to reinfection (Amezcua Vesely et al. 2019). The female genital tract (FGT) is a tolerogenic environment that restricts T cell influx in the absence of inflammation and differs from other mucosal sites, such as the lung, by the absence of secondary lymphoid tissue (Nakanishi et al. 2009). Instead, memory lymphocyte clusters, consisting of CD4 T and B/plasma cells, have been observed in murine uterine tissue months after the infection resolves (Morrison and Morrison 2000). Inducing or boosting these immune populations by mucosal vaccination may be necessary for optimal immunity to CT. Most of the research defining correlates of *Chlamydia* immunity has focused on the female reproductive tract and it is not definitively known if these findings directly translate to other clinically relevant sites like the ocular conjunctiva, rectum, and male urethra/testis. The evidence to date suggests that these correlates likely translate to other mucosal sites. However, additional testing to confirm this translatability in large animal models like pigs or non-human primates would be ideal.

Murine CD4 T cell clones have demonstrated multiple mechanisms of CM inhibition. These include nitrous oxide production, perforin-mediated cytolysis, and T-cell degranulation after major histocompatibility complex (MHC)-II-dependent recognition of infected epithelial cells (Jayarapu et al. 2010). Further investigation revealed an inducible nitric oxide synthase (iNOS)-dependent mechanism and an independent mechanism of clearance that requires Plac8 (Johnson et al. 2012). It is unknown if similar mechanisms occur in humans. Both CM and CT are susceptible to the effects of IFNγ but have evolved different mechanisms for evasion. Human IFNγ activates indoleamine 2,3-dioxygenase that converts tryptophan to N-formyl-kynurenine thereby depleting tryptophan necessary for chlamydial replication (Byrne et al. 1986, Beatty et al. 1993). CT genital strains have evolved to evade this mechanism by encoding a functional tryptophan synthase to convert indoles produced by other microbiota into tryptophan to support their intracellular replication (Fehlner-Gardiner et al. 2002). Murine IFNγ induces expression of p47 GTPases in murine cells that restrict CT growth (Nelson et al. 2005, Bernstein-Hanley et al. 2006). CM resistance to murine IFNγ may be due to the function of the putative inclusion membrane protein TC0574 that could inhibit inclusion destruction by caspases or related pro-death cysteine proteases (Giebel et al. 2019). Humans lack p47 GTPases but possess IFNγ inducible guanylate-binding proteins (GBP) that possess GTPase activity. Data suggest that CT can block ubiquitination by GBPs but remain susceptible to GBP-mediated inflammasome activation (Finethy et al. 2015).

### Platform selection

The first chlamydial vaccines in the 1960s used live or formalin-fixed whole bacteria to prevent ocular infection. These studies demonstrated short-lived protection from active trachoma (Poston et al. 2019). Similar results were obtained using high doses in non-human primates (Kari et al. 2011). There has been recent success in removing pathogenic factors from *Chlamydia spp*. with the goal of optimizing whole-cell-based vaccines. The plasmid encoded pgp3 and chromosomal TC0237/TC0668 were identified as virulence factors that induce hydrosalpinx in CM-challenged mice (Liu et al. 2014, Chen et al. 2015, Huang et al. 2015, Conrad et al. 2016). Strains lacking these genes induced a protective memory response in the absence of oviduct pathology consistent with previous studies using plasmid-deficient CM (O'Connell et al. 2007). These data provide valuable insight into chlamydial pathogenicity but are not likely to overcome safety concerns for use as human vaccines.

The use of inactivated CT EBs may alleviate some safety concerns but has not demonstrated significant protection in mouse models. Although UV-inactivated whole organisms have been used to investigate the induction of protective responses, there are concerns regarding cost, safety, lack of scalability, reproducibility, and poor efficacy in early chlamydial trachoma protection trials (Grayston et al. 1963, Sowa et al. 1969, Grayston and Wang 1978) that preclude their use as commercial vaccines. The short-term protection observed implies an effective vaccine is feasible with the goal of a safe subunit vaccine. Of candidate immunogens that have been investigated to date, the major outer membrane protein (MOMP) is the most studied. Vaccination of female mice with native MOMP isolated from the chlamydial envelope provided significant protection against challenge, determined by lowered cervical burden, accelerated clearance, and reduced oviduct pathology (Pal et al. 2005, Farris et al. 2010). However, these results did not translate with recombinant MOMP (Sun et al. 2009, Tifrea et al. 2011, Pal et al. 2017), suggesting that some of the protection observed was derived from MOMP-conformation-dependent antigen recognition. This confirmation dependency could impact both memory B and T cell induction (Tifrea et al. 2011, Peng et al. 2022). Antibody recognition of conformational epitopes may promote combined neonatal Fc receptor translocation to the subepithelium and antigen-presentation by Fc-receptor expressing professional antigen-presenting cells (Moore et al. 2003, Armitage et al. 2024). This would accelerate a memory CD4 T cell response and may be important for optimal early control and reduced upper genital tract pathology. The T cell response may also be directly affected by antigen conformation. The level of *Yersinia pestis* Caf1 antigen processing and presentation was dependent on the structure of MHC class II binding epitopes (Musson et al. 2006). Epitopes in the globular domain were presented by MHC class II after lysosomal processing, while epitopes in the flexible strand were presented without proteolytic processing. This could have implications for MOMP epitope presentation due to the cysteine residues that form intramonomeric disulfide bonds in the transmembrane region (Yen et al. 2005). Thus, trimeric native MOMP and monomeric recombinant MOMP may load different MHC class II epitopes that could influence the breadth and magnitude of the CD4 T cell response.

A human vaccine with conformationally correct MOMP isolated from live bacteria is not amenable to upscaling for commercial production and would be complicated by multiple serovars expressing different MOMP extracellular variable domains. Attempts to fold recombinantly expressed MOMP in lipid nanodiscs were successful in demonstrating efficacy in an animal model (Tifrea et al. 2021). However, endotoxin contamination remains a concern for nanoparticle vaccine formulations and makes manufacturing and scale-up problematic (Hannon and Prina-Mello 2021, Costa et al. 2023). An approach to bypass the need for conformationally correct recombinant MOMP has been tested in a phase 1 study of CTH522, consisting of MOMP immunorepeat segments spanning the variable domain four region of serovars D, E, F, and G and adjuvanted with liposomal cationic adjuvant formulation (CAF01) or aluminum hydroxide (alum) (Abraham et al. 2019). These formulations were delivered three times via intramuscular injection followed by two unadjuvanted intranasal inoculations and were well tolerated and immunogenic in humans. Disappointingly, significant protection was not observed after the administration of the CTH522/CAF01 vaccine to nonhuman primates (Lorenzen et al. 2022).

Other candidate membrane antigens tested in preclinical vaccine studies include polymorphic outer membrane proteins (Yu et al. 2014) and the adhesin, outer membrane complex B (Olsen et al. 2010, Finco et al. 2011) but none has achieved the level of protection induced by native MOMP. When recombinant Chlamydia protease-like activity factor (CPAF), a secreted protein important in pathogenesis (Paschen et al. 2008), was paired with IL-12 or CpG oligodeoxynucleotides (ODN) (TLR9 agonist) and delivered intranasally, it led to an abbreviated infection and reduced oviduct pathology in mice (Cong et al. 2007, Murthy et al. 2007). Profiling of human antibody responses revealed this antigen to be immunodominant and immunoprevalent in *Chlamydia*-exposed women (Liu et al. 2022). These data indicate this highly conserved antigen is a strong candidate for a human vaccine that may be paired with membrane proteins like MOMP, PmpF, and/or PmpG (Yu et al. 2012, Yu et al. 2014).

Most clinically available vaccines against bacterial pathogens induce antibodies as a primary mode of protection. However, chlamydial protection is dependent on CD4 T cell-mediated responses (Perry et al. 1997, Wang et al. 1999, Yu et al. 2011, Stary et al. 2015, Poston et al. 2017). Until the past decade, generating vaccines against intracellular bacterial pathogens, where cell-mediated immunity is required for protection, was hampered by the lack of safe and effective Th1 and Th17-inducing adjuvants (Lavelle and Ward 2022). Charge-switching synthetic adjuvant particles incorporating the TLR7/8 agonist R848 (resiquimod) complexed to UV-inactivated chlamydial EBs induced significant protection from burden and disease upon vaginal challenge in a female mouse model, demonstrating the power of adjuvants to accelerate induction of robust chlamydial-specific IFNγ+ T cell responses (Stary et al. 2015). Early studies using CPAF as an immunogen demonstrated protection when CPAF was adjuvanted with IL-12 or multiple doses of CpG before and after CPAF immunization (Cong et al. 2007, Murthy et al. 2007). However, the toxicity of IL-12 administration and the implausibility of delivering CpG on consecutive days make these approaches unsuitable for human vaccination.

Adjuvants that target endosomal TLR7 and TLR9 pathways initiate the production of IL-12 and IFNs that induce IFNγ+ CD4 T cells (Wille-Reece et al. 2005, Kastenmuller et al. 2011, Temizoz et al. 2015, Carroll et al. 2016). A single adjuvant may be unable to elicit a broad enough cytokine response sufficient to drive protective T cell-mediated immunity, but two or more agonists can synergistically enhance vaccine efficacy (Temizoz et al. 2015, Kocabas et al. 2020). Stimulator of interferon genes (STING), induces the production of cytokines that drive cell-mediated immunity (Ahn and Barber 2019). Agonists of the cyclic guanosine monophosphate-adenosine monophosphate synthase (cGAS)-STING pathway have emerged as safe adjuvants that activate type I IFNs, pro-inflammatory cytokines, and the inflammasome for IL-1β and IL-18 production (Webster et al. 2017, Ou et al. 2021). These agonists elicit germinal center formation and more potent humoral responses when compared to adjuvants like CpG and shift the response from Th2/IgG1 dominant to a balanced IgG1/IgG2 (Th1) profile (Gray et al. 2012). STING is directly activated by cyclic dimeric adenosine monophosphate (c-di-AMP, CDA), a bacterial metabolite. CDA matures dendritic cells and promotes antigen capture, processing, and presentation for T cell priming, resulting in Th1 and Th1/17 responses (Ebensen et al. 2011, Volckmar et al. 2019). STING agonists have not been tested as vaccine adjuvants against CT but have enhanced host resistance against SARS-CoV-2, *Mycobacterium tuberculosis*, and *Bordetella pertussis*, when administered via parenteral and mucosal routes (Carroll et al. 2016, Allen et al. 2018, Van Dis et al. 2018, Humphries et al. 2021). STING agonists synergize with TLR7 or TLR9 agonists for broader cytokine responses, leading to higher frequencies of antigen-specific IFNγ+ CD4 T cells (Temizoz et al. 2015, Collier et al. 2018, Kocabas et al. 2020, Zhang et al. 2022). A phosphorothioate CDA analog, 2'3'-c-di-AM(PS)2 (Rp,Rp) (ADU-S100), has increased *in vivo* stability and stimulates human and mouse STING alleles (Corrales et al. 2015) and was safe in clinical trials (Meric-Bernstam et al. 2022). The non-nucleotidyl small molecule diABZI had demonstrated increased potency and safety in humans making it one of the most promising STING agonists to date (Ramanjulu et al. 2018, Talley et al. 2021).

Vaccination with CTH522 plus CAF01 induced higher cell-mediated IFNγ responses and more consistent serum antibody titers than CTH522 adjuvanted with alum. CAF01 contains liposomes formed by N,N′-dimethyl-N,N′-dioxtadecylammonium (DDA) with the synthetic mycobacterial immunomodulator α,α′-trehalose 6,6′-dibeheneate (TDB). DDA targets the vaccine antigen to antigen-presenting cells and the TDB induces cytokines that drive a Th1/17 response (Hansen et al. 2008, Wern et al. 2017). This response was demonstrated to be MyD88-dependent but TLR-independent (Agger et al. 2008). Later studies have demonstrated that TDB initiates the inflammatory response through the macropahge inducible Ca2+-dependent lectin receptro (Mincle) receptor and triggers Syk-Card9-dependent APC activation along with the IL-1/MyD88 pathway (Werninghaus et al. 2009, Schoenen et al. 2010, Desel et al. 2013) leading to interferon alpha/beta receptor alpha chain (IFNAR1)-dependent CD4 T cell responses (McEntee et al. 2020). The safety of Mincle agonists in human trials (van Dissel et al. 2014, Abraham et al. 2019, Dejon-Agobe et al. 2019) is encouraging, but the contribution of these responses to protection is not known since a phase II trial has yet to begin. Testing clinically safe Mincle, TLR, and STING agonists alone and in combination may provide a path forward to an effective CT vaccine.

**Figure 1.**
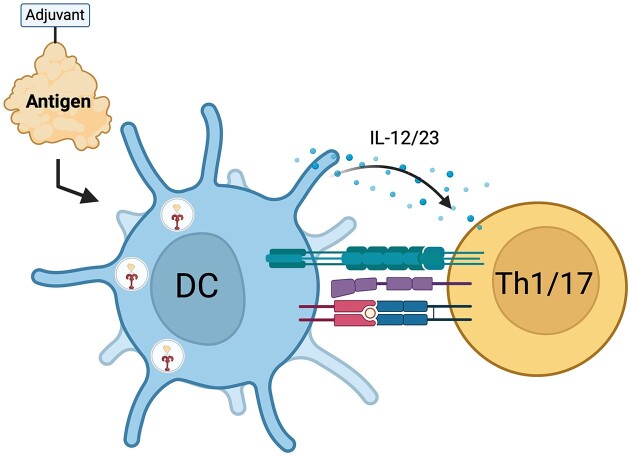
Co-delivery of antigen-adjuvant conjugates enhances dendritic cell-mediated CD4 T cell activation.

Antigen-adjuvant conjugates, or immunogens coupled to synthetic adjuvants, further potentiate the immune response by co-delivering vaccine components to an antigen-presenting cell, providing prolonged antigen presentation that induces adaptive immune responses through intracellular storage and depot effects, and boosting production of immunostimulatory cytokines and chemokines (van Montfoort et al. 2009, Moyle 2017, Xu and Moyle 2018, Ho et al. 2021). (Fig. 1) This approach has superior efficacy in preclinical vaccination studies (Tighe et al. 2000, Wille-Reece et al. 2005, Wille-Reece et al. 2005, Kastenmuller et al. 2011, Zom et al. 2018, Bhagchandani et al. 2021). Furthermore, conjugating these agonists to vaccine antigens facilitates endosomal uptake and increases TLR signaling for APC activation, which reduces the amount of adjuvant required for immunogenicity and lowers reactogenicity (Oh and Kedl 2010, Irie et al. 2020, Weiss et al. 2021, Zhang et al. 2022). Future vaccine strategies could explore the incorporation of TLR7/9, Mincle, and STING agonists alone and in combination with adsorption or bioconjugation strategies to further enhance immunogenicity by upregulating inflammatory cytokines and type I interferons.

Viral vectors are an efficacious delivery system for immunization that provides expression of antigens within target cells. These vectors are usually live-attenuated or replication-deficient, which allows for the preservation of immunogenicity while reducing pathogenicity. They have provided a proof-of-concept approach for preclinical assessment of putative antigens and profiling correlates of immune protection. Some viral vector vaccines that have progressed to clinical trials and/or licensing include recombinant canarypox (ALVAC-Human immunodeficiency virus), Modified Vaccinia Ankara (MVA)-E2 (human papillomavirus), recombinant vesicular stomatitis (VSV)-Zaire ebola virus (ZEBOV), chimpadenovirus (ChAdOx1), and recombinant Ad26 for respiratory syncytial virus (RSV) and Zika virus (ZIKV) infections (Rerks-Ngarm et al. 2009, Rosales et al. 2014, Dahlke et al. 2017, Williams et al. 2020, Ewer et al. 2021, Pollard et al. 2021, Salisch et al. 2021). Viral vector vaccines have the benefit of inducing a broad adaptive immune response characterized by high frequencies of CD4 T cells, cytotoxic CD8 T cells (CTLs), and class-switched antibody and memory B cells. Recombinant adenoviruses have demonstrated the most utility to date due to their high transduction efficiency, antigen expression, and broad tropism. These vectors have been licensed as SARS-CoV-2 vaccines and include Vaxzeveria (ChAdOx1 nCoV-19/AZD1222), Janssen COVID-19 vaccine (Ad26.COV2.S), and Sputnik V (Gam-COVID-Vac). MVA is also a promising vector derived from the highly attenuated Ankara strain. This vector is licensed as a vaccine for smallpox and has demonstrated safety and efficacy in human trials (McShane et al. 2004, de Vries et al. 2018, Chiuppesi et al. 2022). It has commonly been used in heterologous prime-boost regimens with adenoviral vectors against multiple pathogens (Rampling et al. 2016, Tapia et al. 2016) and the heterologous combo of MVA BN-Filo and Ad26-ZEBOV is licensed for human use against Ebola virus disease (Pollard et al. 2021).

Viral vector vaccines proven safe in humans provide an opportunity to test recombinant chlamydial antigens using this platform due to their ability to induce Th1 responses. To date, there are limited studies investigating viral vectored chlamydial vaccines in animal models. Intranasal delivery of influenza-vectored MOMP epitopes inserted into the neuraminidase stalk region induced durable MOMP-specific Th1 responses in the spleen and draining lymph nodes (He et al. 2007). This was associated with reduced burden after intravaginal challenge with CT serovar D. Intranasal administration of adenovirus-vectored CPAF (AdCPAF) elicited a balanced CPAF-specific IFNγ response between CD4 and CD8 T cells in C57BL/6, C3H/HeN, and BALB/c mice after a single dose (Brown et al. 2012). Mice receiving a heterologous regimen with AdCPAF and recombinant CPAF adjuvanted with CpG and the immunomodulatory peptide HH2 demonstrated a greater Th1 response compared to a mixed Th1/17 response following homologous rCPAF prime-boost. Both prime-boost regimens reduced the frequency of hydrosalpinx by 50% in C3H/HeN mice compared to single-dose AdCPAF administration. The use of hAd5-MOMP and MVA-MOMP in a prime-boost strategy also incorporating DNA-MOMP and recombinant MOMP plus CAF01 has recently been investigated in cynomolgus macaques (Lorenzen et al. 2022). The regimen utilizing DNA-MOMP as the priming dose followed by CAF01 adjuvanted CTH522 elicited a more balanced CD4/CD8 response and abbreviated the duration of infection.

The use of mRNA technology as a vaccine platform has made major strides. The mRNA that encodes the selected immunogen is encapsulated in lipid particles for protection against degradation and delivery into host cells where the mRNA is translated into protein. This mRNA and liposome combination provides an intrinsic adjuvant effect, allows for repeated administration, and avoids pre-existing immunity associated with some viral vectors (Verbeke et al. 2022, Xie et al. 2023). This platform has consistently demonstrated safety in humans, comes with no risk of insertional mutagenesis, and has the benefit of rapid, low-cost manufacturing. This was recently demonstrated with the rapid turnaround of the Pfizer Tozinameran (BNT162b2) and Moderna mRNA-1273 vaccines against SARS-CoV-2. These vaccines were produced in an *in vitro* cell-free transcription reaction that expedites the conventional vaccine manufacturing process. Both were the first mRNA vaccines licensed for humans and generated both neutralizing antibodies and Th1-biased responses (Polack et al. 2020, Baden et al. 2021). This platform has been effectively utilized against SARS-CoV-2 with additional vaccines against influenza, RSV, and ZIKV in clinical trials (Feldman et al. 2019, Aliprantis et al. 2021, Essink et al. 2023). Preclinical research studies are investigating the efficacy of mRNA vaccines against CT and other bacterial pathogens (Maruggi et al. 2017, Kon et al. 2023, Pine et al. 2023).

### Route of immunization

**Figure 2.**
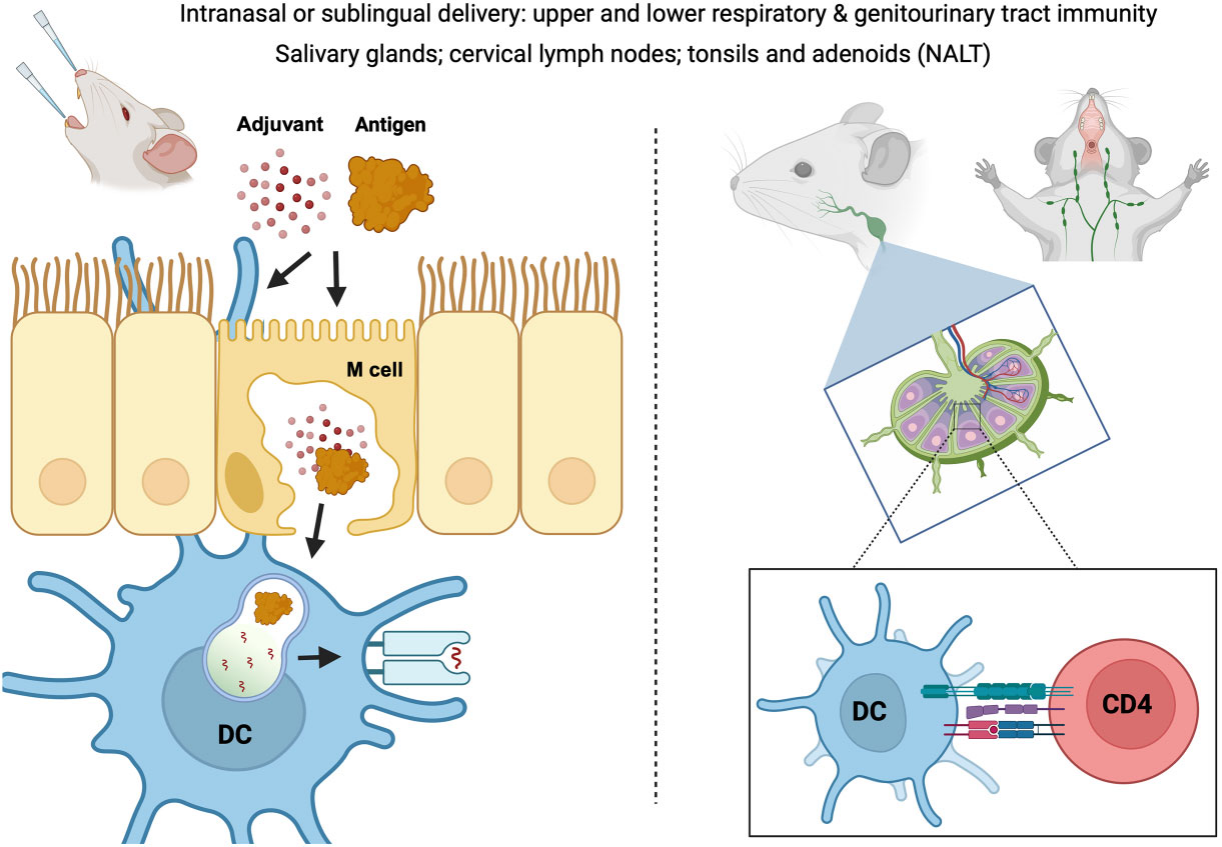
Intranasal and sublingual immunization activates submucosa dendritic cells (left) that migrate to the draining cervical lymph nodes and activate antigen-specific CD4 T cells (right) that provide transmucosal immunity.

Current FDA-approved mucosal vaccines use inactivated or live-attenuated pathogens with only one intranasal vaccine to date (FluMist®) with the rest targeting gut pathogens and are delivered orally. Subunit vaccines are safe, stable, and highly manufacturable, but have exhibited poor immunogenicity when given intranasally mainly due to the challenges of delivery. Vaccine uptake in the underlying mucosal immune compartment is hindered by proteolytic enzymes, acidic conditions, mucociliary clearance, and the lack of diffusion across the epithelial monolayer. These are barriers to effective antigen delivery to the lamina propria and draining lymph nodes where dendritic cells capture and present antigens to CD4 T cells. (Fig. 2) Priming of CD4 T cells in the nasal-associated lymphoid tissue imprint chemokine receptors and integrins like CCR10 and α4β1 that allow effector CD4 T cells to home to both the respiratory and genitourinary tracts (Holmgren and Czerkinsky 2005). Intranasal vaccination using TLR agonist-adjuvanted inactivated *Chlamydia* achieved superior genital tract protection in mice compared to parenteral routes (Murthy et al. 2007, Stary et al. 2015). Every chlamydial vaccine tested preclinically has achieved superior results with mucosal administration when compared directly to systemic administration (de la Maza et al. 2021). However, the field has lacked safe mucosal adjuvants until recently. Many adjuvants are too reactogenic when delivered systemically or cause nasal irritation. However, the issues of reactogenicity and delivery may be overcome with sublingual (s.l.) delivery using tablets (Kelly et al. 2022) or thermoresponsive gels (White et al. 2014, Lal et al. 2017). These formulated sublingual vaccines enhance contact time between antigen and the oral mucosa and s.l. delivery of MOMP adjuvanted with CTA1-DD (fusion protein of cholera toxin A1 subunit and immunoglobulin binding domain from *Staphylococcus aureus* protein A) led to a reduced incidence of oviduct pathology in mice challenged with CM (O'Meara et al. 2014). Multiple delivery approaches are being explored, opening the door for the practical development of mucosal delivery via intranasal and sublingual routes (Huang et al. 2022) that provide genital immunity. Oral immunization with live-attenuated *Chlamydia* (Wang et al. 2018, Zhou et al. 2022, Wang et al. 2023) and a MOMP vaccine adjuvanted with cholera toxin, CpG-ODN, and Lipid C have also demonstrated protection in mice (Hickey et al. 2010). Covalently coupling protein antigens with FcRn-binding moieties or in liposomes may facilitate better transportation across the mucosal epithelium resulting in enhanced antigen-specific T- and B-cell responses (Lu et al. 2011, Ye et al. 2011, Olsen et al. 2015, Tada et al. 2018, Kumar et al. 2021, Ochsner et al. 2021, Hartwell et al. 2022, Li et al. 2023, Ozberk et al. 2023). Mucosal vaccination offers advantages over systemic immunization that include avoiding needle fears, the potential for self-administration, and induction of mucosal and systemic immunity.

### Clinical testing

The field seeks to develop an efficacious CT vaccine to prevent male and female genital tract disease with the larger goal of preventing sexual transmission. A vaccine would ideally target adolescents before sexual debut to establish immunity prior to exposure. Human clinical trials for efficacy will require the enrollment of sexually active adults with a target age of 18–25 years since CT is most prevalent in this age group (Miller et al. 2004). Both cis- and transgender males and females would be eligible for enrollment, and efficacy would be assessed by nucleic acid amplification testing (NAAT) of clinically obtained samples. Reductions in the frequency of infected individuals and bacterial burden in those who become infected could serve as primary endpoints in a clinical trial. Based on modeling studies, a CT vaccine that achieves at least a 50% reduction in the rate of infection for 10 years will decrease infection prevalence by 50% after 13 years and essentially eradicate the infection in 24 years (de la Maza and de la Maza 1995). A high-coverage vaccine that reduces the chlamydial peak load by 2-log_10_ could eradicate a chlamydial epidemic in ~20 years (Gray et al. 2009). Thus, a 50% effective vaccine that results in a 2-log_10_ reduction in burden among those who acquire infection relative to individuals in the unvaccinated arm would be highly beneficial by decreasing the prevalence of infection and reproductive morbidities like PID and infertility.

The use of reproductive tract disease as a secondary clinical endpoint is influenced by multiple factors. The clinical diagnosis of PID is insensitive and nonspecific with multiple causes. Although CT causes ~one-third of PID cases, determining causation would prove difficult, and infertility may not be recognized for years after prior CT infection(s). The previously executed laparoscopic diagnosis is invasive and no longer routinely performed. Determining more precise and practical measures of upper genital tract inflammation and disease is a priority for the design of practical clinical trials. Future work will seek to determine the role of techniques like MRI and power Doppler along with the potential for blood biomarkers for less invasive sampling (Zheng et al. 2018, Zheng et al. 2018). Endometrial biopsies are a minimally invasive procedure used to determine upper genital tract infection and inflammation. However, a high cervical bacterial burden may be an acceptable surrogate for upper genital infection (Russell et al. 2016). A non-invasive biomarker for upper genital tract ascension would be a powerful tool to use in conjunction with NAAT testing. This tandem approach would help determine if a decrease in infection parallels a decrease in disease and determine if PID is prevented in vaccinated individuals who become infected. It would also address if breakthrough infection led to an enhanced risk for PID. This could also have important implications in determining vaccines that may enhance disease. For example, viral vector vaccines with the potential to elicit high frequencies of antigen-specific CD8 T cells, which may not effectively reduce *Chlamydia* burden, could potentially enhance immunopathology because of ongoing stimulation during chronic infection in the absence of protective CD4 T cells. Future research aims to address better ways of measuring upper genital tract infection, inflammation, and disease for the purpose of clinical vaccine testing.

## Conclusion

Major progress has been made in *Chlamydia* vaccinology to identify the importance of CD4 T cell immunity and the potential for resident memory populations to enhance protection. Recent advancements in mucosal adjuvant development provide a path forward for developing a vaccine that is safe and well-tolerated in humans. Pre-clinical testing of these novel vaccine formulations in mice will help determine efficacy and safety against genital infection and disease. This will help triage vaccines for further testing in non-human primates prior to Phase 1 clinical trials. A partially protective vaccine would significantly reduce infection prevalence. Current research efforts will continue to identify vaccines suitable for human translation and identify correlates of protective immunity versus pathogenic responses.
